# In Vitro Interaction of Lithium on Phospholipids in Human Erythrocytes

**DOI:** 10.1080/15376510701623961

**Published:** 2008-06-23

**Authors:** A. Sankiewicz, E. Gorodkiewicz, Z. Figaszewski

**Affiliations:** Department of Electrochemistry, Institute of Chemistry, University of Bialystok,Al.J.Pilsudskiego11/4, 15–443 Bialystok, Poland; Department of Electrochemistry, Institute of Chemistry, University of BialystokAl.J.Pilsudskiego1 1/4, 15–443 Bialystok, Poland; Laboratory of Electrochemical Power Sources, Faculty of Chemistry, University of Warsaw,Pasteur St. 1, 02–093 Warsaw, Poland

**Keywords:** Human Erythrocytes, Lithium Carbonate, Phospholipids

## Abstract

Lithium salts are used in the treatment of mania and as prophylaxis against manic depressive disorder. The aim of these studies was the in vitro investigation of the effect of lithium on phospholipids of human erythrocyte membranes. Erythrocytes were treated with lithium for 1 h. Phospholipids phosphatidylinositol (PI), phosphatidylserine (PS), phosphatidylethanolamine (PE), and phosphatidylocholine (PC) were separated from erythrocyte ghosts and determined by HPLC. Blood samples from healthy adults were investigated. A very strong decrease in PC content in erythrocyte membranes due to lithium in vitro treatment was found, as well as a statistically significant increase in PI content.

## INTRODUCTION

Lithium salts are used in the treatment of mania and as prophylaxis against manic depressive disorder (Goodwin and Jamison 1990). Lithium is normally present in the body at trace concentrations and is toxic at higher concentrations. In therapeutic treatment, it is dosed in concentrations below 2 mM (Romano et al. 1995). A long period of treatment with lithium causes lesions to the gastroenteric tube, the nervous system, and kidneys. The therapeutic activity of lithium is supposed to be caused by lithium inhibition of inositol phosphatases, especially the inositol monophosphatase (Manji et al. 1995). Lithium also reduces brain concentrations of prostaglandin E_2_ and cyclogenase 2 (Rapoport and Bosetti 2002), as well as decreases phospholipase A_2_ activity (Rapoport and Bosetti 2002; Chang and Jones 1998). Most investigations concerning the lithium effect explain its therapeutic effect. However, the high ability of lithium to inhibit numerous enzymes may also be a reason for unexpected side effects.

## EXPERIMENTAL

HPLC analysis was done with a Merck Hitachi liquid chromatograph with a diode array detector (model L-4500, Tokyo Japan). A Superspher Si 60 column (250 x 4 mm) was applied. Phospholipids standards-phosphatidylinositol (porcine liver), phosphatidylserine (bovine brain), phosphatidylethanolamine (porcine liver), and phosphatidylcholine (porcine liver) (Larodan AB, Sweden) were used. Butylated hydroxytoluene (BHT) (analytical grade Sigma-Aldrich) as well as sodium phosphate buffer-1 (osmolality 310 mmol/kg^−1^, pH = 7.4) and sodium phosphate buffer-2 (osmolality 20 mmol/kg^−1^ pH = 7.4), acetonitrile, methanol, n-hexane, and isopropanol (all gradient grade for chromatography, Merck) were used. Other reagents including lithium carbonate were of analytical grade. Water was deionized with a MiliQ(Milipore) apparatus. All solvents in the experiment were deoxidized with a Polsonic ultrasonic cleaner.

**FIGURE 1:**
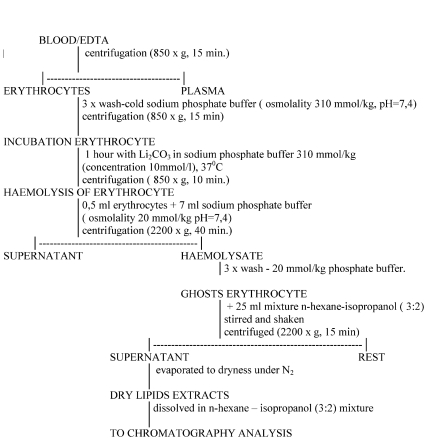
Isolation of erythrocyte membranes and phospholipid extraction.

Blood samples taken from eight different healthy adult subjects were supplied by the Blood Donor Centre of Bialystok. The age of the donors varied between 20 and 50 years. Blood was treated with EDTA as an anticoagulant. The procedural sequence of isolation of erythrocyte membranes and extraction of phospholipids was performed in accordance to Radin (1981) and Ostrowska et al. (2000) and is given in Figure 1. The erythrocytes were incubated at 37°C for 1 h with 10 mM lithium carbonate in the phosphate buffer-1. To prevent the oxidation of phospholipids during extraction, 0.03% of BHT was added, along with flushing with nitrogen at each step of the procedure. Separation and determination of isolated phospholipids were performed by normal phase HPLC with iso-cratic elution. The mobile phase was acetonitrile-methanol-85% phosphoric acid mixture of 130:5:1.5 volume ratio. The flow rate of the mobile phase was 1 mL/min^−1^. The chromatogram was recorded at 214 nm wavelength (Ostrowska et al. 2000). The peaks of phosphatidylinositol (PI), phosphatidylserine (PS), phosphatidylethanolamine (PE), and phosphatidylcholine (PC) are well separated. Unfortunately, sphingomyelin is not determined under the selected conditions.

### Statistical Analysis

The results are expressed as mean values ± SD (n = 8). Statistically significant differences were determined by the Student's *t*-test, as well as ANOVA. *p* ≤ 0.05 was considered statistically significant.

## RESULTS AND DISCUSSION

**FIGURE 2:**
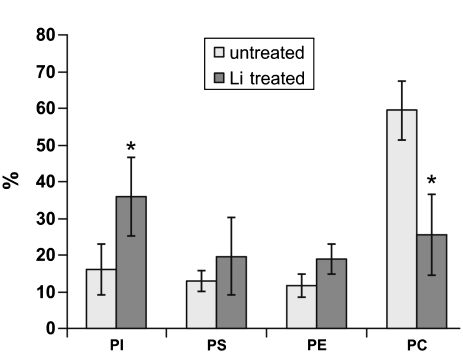
Comparison of the phospholipid content of erythrocyte membranes as a percentage of the total phospholipids from Li^+^-treated and Li^+^-untreated (control). * Statistically significant differences for p ≤ 0.05 with respect to control.

These studies investigate the effect of lithium on phospholipids of human erythrocyte membranes. Concentrations of lithium above the therapeutic dose by half an order of magnitude and in vitro experiments were undertaken. Results are shown in Figure 2. The results clearly indicate the statistically significant decrease in PC content in erythrocytes due to lithium in vitro treatment. This effect was predicted by Jenden et al. (1980) and Hanin et al. (1980), but not observed in in vivo experiments with persons treated with lithium (Pleul and Müller-Oearlinhausen 1986; Bramham and Riddell 1995). Jenden et al. (1980) and simultaneously Hanin et al. (1980) discovered 10 to 30 times higher choline levels in erythrocytes treated with lithium and predicted that choline originates from a breakdown of phosphatidylcholine. The reason for the difference between in vitro and in vivo experiments is probably significantly lower lithium concentrations in the in vivo experiments and complex reactions involving lithium.

The other result of the research is an increase in PI content. Although it is a surprising effect, the increase is statistically significant. A similar effect is observed in neuroblastoma membranes during chronic Li^+^ treatment for 4 to 6 weeks (Layden et al. 2005). This may be caused by the inhibition of PI phosphorylation to PIP (phosphatidylinositol-4-phosphate) and subsequently to PIP2 (phosphatidylinositol-4,5-bisphosphate), which is the next stage in the PI cycle (Shaladubina at al. 2001). This is why PI is accumulated in membranes, since the synthesis of PI is not inhibited but PI transformation is stopped. Probably the inositol accumulation process could be connected with the phosphatidilcholine decrease. Phos-phatidilcholine undergoes hydrolysis into phosphatidic acid and free choline. Then phosphatidic acid changes into the form of diacylglicerol (DAG). DAG is converted into cytidine diphosphate-diacylglycerol (CDP-DAG). CD-DAG could react with inositol to form phosphatidylinositol (PI) (Shaladubina et al. 2001; Murray et al. 1996).
